# Evolving DNA methylation and gene expression markers of B-cell chronic lymphocytic leukemia are present in pre-diagnostic blood samples more than 10 years prior to diagnosis

**DOI:** 10.1186/s12864-017-4117-4

**Published:** 2017-09-13

**Authors:** Panagiotis Georgiadis, Irene Liampa, Dennie G. Hebels, Julian Krauskopf, Aristotelis Chatziioannou, Ioannis Valavanis, Theo M.C.M. de Kok, Jos C.S. Kleinjans, Ingvar A. Bergdahl, Beatrice Melin, Florentin Spaeth, Domenico Palli, R.C.H. Vermeulen, J. Vlaanderen, Marc Chadeau-Hyam, Paolo Vineis, Soterios A. Kyrtopoulos, on behalf of the EnviroGenomarkers consortium, Ralph Gottschalk, Danitsja van Leeuwen, Leen Timmermans, Maria Botsivali, Benedetta Bendinelli, Rachel Kelly, Lutzen Portengen, Fatemeh Saberi-Hosnijeh, Göran Hallmans, Per Lenner, Hector C. Keun, Alexandros Siskos, Toby J. Athersuch, Manolis Kogevinas, Euripides G. Stephanou, Antonis Myridakis, Lucia Fazzo, Marco De Santis, Pietro Comba, Hannu Kiviranta, Panu Rantakokko, Riikka Airaksinen, Päivi Ruokojärvi, Mark Gilthorpe, Sarah Fleming, Thomas Fleming, Yu-Kang Tu, Bo Jonsson, Thomas Lundh, Wei J. Chen, Wen-Chung Lee, Chuhsing Kate Hsiao, Kuo-Liong Chien, Po-Hsiu Kuo, Hung Hung, Shu-Fen Liao

**Affiliations:** 1 Institute of Biology, Medicinal Chemistry and Biotechnology, National Hellenic Research Foundation, 48, Vassileos Constantinou Avenue, 11635 Athens, Greece; 2 Department of Toxicogenomics, Maastricht University, 6229 Maastricht, ER Netherlands; 3 Department of Biobank Research, and Occupational and Environmental Medicine, Department of Public Health and Clinical Medicine, Umeå University, 901 87 Umeå, Sweden; 4 Department of Radiation Sciences, Oncology, Umeå University, 901 87 Umeå, Sweden; 5 The Institute for Cancer Research and Prevention, 50141 Florence, Italy; 6 Institute for Risk Assessment Sciences, Utrecht University, Utrecht, Netherlands; 7 Department of Epidemiology and Biostatistics, MRC-HPA Centre for Environment and Health, School of Public Health, Faculty of Medicine, Imperial College, London, W2 1PG UK

**Keywords:** Epigenomics, Transcriptomics, miRNA, Biomarkers of risk, Molecular epidemiology, Prospective cohort

## Abstract

**Background:**

B-cell chronic lymphocytic leukemia (CLL) is a common type of adult leukemia. It often follows an indolent course and is preceded by monoclonal B-cell lymphocytosis, an asymptomatic condition, however it is not known what causes subjects with this condition to progress to CLL. Hence the discovery of prediagnostic markers has the potential to improve the identification of subjects likely to develop CLL and may also provide insights into the pathogenesis of the disease of potential clinical relevance.

**Results:**

We employed peripheral blood buffy coats of 347 apparently healthy subjects, of whom 28 were diagnosed with CLL 2.0–15.7 years after enrollment, to derive for the first time genome-wide DNA methylation, as well as gene and miRNA expression, profiles associated with the risk of future disease. After adjustment for white blood cell composition, we identified 722 differentially methylated CpG sites and 15 differentially expressed genes (Bonferroni-corrected *p* < 0.05) as well as 2 miRNAs (FDR < 0.05) which were associated with the risk of future CLL. The majority of these signals have also been observed in clinical CLL, suggesting the presence in prediagnostic blood of CLL-like cells. Future CLL cases who, at enrollment, had a relatively low B-cell fraction (<10%), and were therefore less likely to have been suffering from undiagnosed CLL or a precursor condition, showed profiles involving smaller numbers of the same differential signals with intensities, after adjusting for B-cell content, generally smaller than those observed in the full set of cases. A similar picture was obtained when the differential profiles of cases with time-to-diagnosis above the overall median period of 7.4 years were compared with those with shorted time-to-disease. Differentially methylated genes of major functional significance include numerous genes that encode for transcription factors, especially members of the homeobox family, while differentially expressed genes include, among others, multiple genes related to WNT signaling as well as the miRNAs miR-150-5p and miR-155-5p.

**Conclusions:**

Our findings demonstrate the presence in prediagnostic blood of future CLL patients, more than 10 years before diagnosis, of CLL-like cells which evolve as preclinical disease progresses, and point to early molecular alterations with a pathogenetic potential.

**Electronic supplementary material:**

The online version of this article (10.1186/s12864-017-4117-4) contains supplementary material. Note to Editor: If Journal regulations permit, the Supplementary material should be available to all without any additional authorisation.

## Background

B-cell chronic lymphocytic leukemia (CLL) is the most common adult leukemia in the Western world, accounting for roughly 30% of all leukemias and with incidence rates in different countries ranging between <1 and 5.5 cases per 100,000 [1]. It is characterized by the presence of large numbers (>5000/μl) of clonal, mature B-cells with increased proliferation and prolonged survival and expressing a number of specific surface markers, including CD5, CD19 and CD23. The disease often follows an indolent course, with many patients having no symptoms at diagnosis or surviving for decades after diagnosis without any need for treatment [2]. Nevertheless, despite great progress in therapeutic protocols, CLL remains an incurable disease whose etiology is largely unknown, and some patients have recurrent relapses requiring several lines of treatment [3].

It is well established that CLL is preceded by monoclonal B-cell lymphocytosis (MBL) [4], an asymptomatic hematological condition characterized by the presence of small clones of B-cells, whose prevalence in the general population ranges from less than 1% to nearly 20%, depending on the sensitivity of the detection methods employed [5]. Τwo types of the MBL are recognized, a) low-count MBL (<500 clonal B-cells /μl), which appears to remain stable with near-zero risk of progression to CLL, and b) high-count (or clinical) MBL (500–5000 clonal B-cells /μl), which is believed to be a precursor of CLL with 1–2% of its carriers progressing to clinically defined CLL per year. Currently it is not known what causes some MBL carriers to progress to CLL, and the number of clonal B-cells appears to constitute the best marker predictive of such progression [6, 7]. A number of biomarkers which are present in CLL cells and have prognostic value in relation to the clinical progression or therapeutic response of the disease (e.g. levels of expression of CD38 or ZAP-70, deletions in chromosomes 11, 13 and 17, trisomy 12, mutations in the IgG hypervariable region, mutations in CLL driver genes such as *NOTCH1*, *BIRC3* or *SF3B1*) are also observed in MBL and help to identify MBL patients who are more likely to ultimately develop advanced CLL affecting survival [8, 9]. However such markers of prognosis appear to be of limited value with regard to the prediction of the risk of MBL subjects making the transition to a CLL phenotype [10, 11].

During the past few years a number of studies reported on the ability of various biomarkers measured in the blood of apparently healthy subjects to predict the risk of future diagnosis of CLL [12–16]. These predictive biomarkers concern cell surface markers and mitochondrial DNA copy number and provide limited insight into cellular processes that precede clinical disease. On the other hand, we recently reported that a gene expression profile measured in peripheral blood leukocytes could identify with high accuracy individuals who were diagnosed with CLL 2–17 years later [17] and highlighted genes whose expression was deregulated long before disease diagnosis. In extended analyses we showed that these gene expression profiles showed marked overlap with expression profiles of clinical CLL samples [18] suggesting that circulating cells long before diagnosis harbor CLL-traits. A common limitation of these studies lies in the lack of information regarding the hematological status of the study subjects at recruitment, including the presence or not of undiagnosed CLL. Here we report on the genome-wide epigenomic (DNA methylation) and miRNA expression profiles in peripheral blood leukocytes of the same population as in our above-mentioned study and the identification of prediagnostic epigenetic profiles which predict with high efficiency the risk of future CLL. Importantly, in the current study we utilized DNA methylation profiles, obtained using the Illumina HumanMethylation450 platform, to estimate for each subject the proportions of different subpopulations of white blood cells (WBC) and were therefore able to derive CLL risk-associated profiles, including a revised genome-wide gene expression profile, adjusted for WBC composition. The results reported here offer insights into the evolution of CLL and provide a basis for the development of improved prediagnostic markers predictive of the risk of future development of CLL.

## Methods

### Population

The study was conducted in the context of the European EnviroGenomarkers project [19] and involved subjects from the European Prospective Investigation into Cancer and Nutrition study (EPIC-ITALY) and the Northern Sweden Health and Disease Study (NSHDS) (Table 1). Both studies used population-based recruitment with standardized lifestyle and personal history questionnaires, anthropometric data and blood samples collected at recruitment (1993–1998 for EPIC-ITALY; 1990–2006 for NSHDS). Buffy coats were isolated from the collected blood samples and placed in long-term cold storage. Although the EnviroGenomarkers project was originally designed as two nested case-control studies, one for B-cell lymphoma and one for breast cancer [17], in the presently reported study subjects who eventually developed disease other than CLL were excluded, leaving 28 subjects who developed CLL (cases) and 319 subjects who remained apparently healthy until the end of the observation period (controls). No participant was diagnosed with disease within less than 2 years of blood sample collection and for this reason all participants were treated as apparently healthy at recruitment. In order to minimize the effects of sample handling on the omic profiles, subjects were included in the current study only if, at recruitment, the processing of their blood samples and freezing of the buffy coats had been completed within 2 h of collection [20].

**Table 1:** Demographic characteristics of the study population. DNA methylation profiles were available for all subjects, while gene and miRNA expression profiles were available as indicated in the Table

	Total	Cases	Controls
Total (N)	347	28	319
EPIC Italy; *N* (% of total)	133	9	124
NSHDS; *N* (% of total)	214	19	195
Males; *N* (% of total)	135	18	117
Females; *N* (% of total)	212	10	202
Age; mean (SD)	52.3 (7.7)	52.0 (8.1)	52.3 (7.7)
BMI; mean (SD)	26 (4.1)	25.6 (3.3)	26.0 (4.1)
Smokers (%)	72 (2.1%)	2 (7.1%)	70 (2.2%)
Subjects with transcriptomic profile (*N*)	307	25	282
Subjects with miRNA expression profile (*N*)	111	11	100

### Estimation of WBC from DNA methylation profiles

WBC composition was estimated using a published algorithm [21] and DNA methylation data derived from purified normal blood cell sub-populations (CD4-, CD8-, T- and NK cells, monocytes, granulocytes) publicly available in the *FlowSorted.Blood.450 k* Bioconductor package, as previously described [22]. The applicability of this methodology to the estimation of the WBC composition in CLL patients was recently confirmed [23]. To evaluate further the reliability of this method with CLL blood we applied it to published 450 k methylation data of purified (>95%) CLL cells isolated from the blood of 139 patients as well as 26 samples of normal B-cells, available from the International Cancer Genome Consortium [24]. The proportions of B cells, as estimated via DNA methylation, showed a single distribution as evaluated by the maximum BIC criterion, with a mean value of 88.5% (variance = 0.4%; SD 6.6%), while all other cell types gave mean values below 1.8% except CD4 which gave a mean value of 7.1% (results not shown). The accuracy of these estimates is comparable to that exhibited by the same methodology in normal blood [21].

### Analytical procedures and data processing

RNA and DNA extraction from buffy coats, genome-wide analysis of gene expression (Agilent 4 × 44 K human whole genome microarray platform), CpG methylation (Illumina Infinium HumanMethylation450 platform) and miRNA expression profiling [Agilent Human miRNA Microarray (Release 19.0, 8x60K), representing 2006 human miRNAs], were conducted as previously described [17, 20, 25]. Methylation data were preprocessed initially with GenomeStudio (version 2011.1) Methylation module (version 1.9; Illumina). Subsequently, data normalization to address the issue of unwanted technical variation was performed, using scripts written and ran in MATLAB environment (Mathworks, Release 2012b), making use of the DNA methylation measured in multiple replicates of a technical control sample randomly distributed among the study samples and utilising procedure involving two successive steps of intensity-based correction (a) within-chip and b) across all probes) as previously described [26]. Probes with background signal (*p* < 0.01) in more than 10% of the samples were filtered out. Probes containing SNPs at a distance less than 3 nucleotides from the interrogated CpG cytosine and minor allele frequency > 10% were also omitted as well as probes giving mean methylation for all samples in the range 0- 4% or 96–100%. Missing values imputation (k-nearest neighbor) was applied to the resulting final number of 396,808 target CpG sites. Methylation levels were expressed as M-values corresponding to the logarithmic ratio of the methylated versus the unmethylated signal intensities.

All unsupervised analyses (PCA, clustering) were performed using the denoised signals, correcting for batch effect (date of chip analysis for the epigenetics and date of hybridization for transcriptomics), gender, cohort and smoking status. Use of the batch removal processes built in the Combat in R (version 3.0.2) and the ArrayStudio (Omicsoft, Cary, NC, USA, version 8.0.1.32) software packages gave very similar results, and consequently the batch removal tool of Arraystudio was adopted for further analyses.

### Statistical analyses

Generalized linear models (GLM) using the batch-corrected signals (date of chip analysis for the DNA methylation and date of hybridisation for gene expression data), as well as Linear Mixed Models (LMM) using as random variables those mentioned above, were applied using the ArrayStudio software package. M values for DNA methylation or log2 intensities of mRNA or miRNA expression were the dependent variables, CLL status the independent variable, while as confounder variables we included sex, age, BMI, cohort as well as the six cell type fractions (CD4, CD8, NK cells, monocytes, B-cells, granulocytes). GLM and LMM gave very similar results and consequently, in order to reduce the possibility of overfitting, the GLM was finally adopted and five out of the total six of the cell type fractions (excluding granulocytes) were included as confounders. The estimated adjusted effect sizes are expressed as the least square means (LSM) (also known as EMM - estimated marginal means) which, in an analysis of covariance model, correspond to the group means after having controlled for a covariate [27]. The LSM β values were derived from the corresponding estimated LSM M values and the equation β = 2^M^/(1 + 2^M^). Multiple testing was accounted for with high stringency by using Bonferroni or FDR Benjamini-Hochberg correction.

The selection of CpGs with minimal variation between WBC subtypes was based on the data by Jaffe and Irizarry [22], selecting sites which fulfill the following criteria: a) *p* > 0.00012 (1000fold greater than the raw *p*-value corresponding to Bonferoni-corrected *p* < 0.05) and b) coefficient of variation (CV) <5% across all leukocyte subpopulations (CD4-, CD8-, T- and NK cells, monocytes, granulocytes). PCA was performed using the denoised values of 1308 out of the 10,785 CLL risk-associated CpG (FDR < 0.05) and 1308 CpGs with FDR >0.8 (control) which fulfill the above mentioned criteria. The CV of the CLL risk- associated CpG sites thus selected for the PCA analysis ranged 0.13–5% (mean = 1.96%).

Non-negative matrix factorization (NMF) was performed in Arraystudio using the default parameters maximum iteration *n* = 100 and stopping rule 1X10^−6^, specifying the number of clusters as 2–4.

### Bioinformatics analysis

Gene functional classification analysis was performed using the DAVID bioinformatics tool [28] (default DAVID values, low stringency criteria). Functional analysis of genes associated with DM sites or DE probes was performed using the BioinfoMiner web application, which enables systemic, functional interpretation of omic datasets through the exploitation of various biomedical ontologies, extracting highly enriched gene sets that form cross-talking functional clusters [29]. BioinfoMiner initially maps the input omic data at the gene level and subsequently, through a combination of advanced statistical and network topological criteria, probabilistically prioritizes the resulting genes according to their functionality by comparison with enrichments of random resamplings, thus facilitating the identification and rejection of false positives. The non-parametric, empirical nature of this prioritization approach permits its generic, broad applicability even to classes of statistical testing problems that deflect from traditional hypotheses, as is the case for DNA methylation profiles, ensuring robust performance. Pathway analysis with this tool exploits variations of the StRAnGER and GOrevenge algorithms [30], so that molecular information (functions, processes, cellular compartments) is highlighted according to multiple criteria (enrichment score, expression etc.) while in addition regulatory hub genes which play a pivotal role in the phenotype under study are identified. Differentially methylated or expressed genes were used as input to identify statistically significantly over-represented terms from four different ontologies: Gene Ontology, Human Phenotype Ontology, MGI Mammalian Phenotype Ontology, as well as Reactome pathways Ontology. For the KEGG pathways analysis part the original StRAnGER2 web service was used [29].

### ROC analysis

A variety of classification algorithms were tested for their performance, including SVM with linear kernel, SVM with Gaussian kernel, Bayesian generalized linear regression, Naive Bayes classifier, random forest and k-NN optimized in two different sets of k options. These classifiers were trained on a subset of our data corresponding to 50% of the study subjects (“training set”). To eliminate the effect of the severely unbalanced training set with respect to the proportion of classes (8.5% cases, 91.5% controls), we implemented the ROSE algorithm from the ‘ROSE’ R package [31]. Thus we trained the classifiers using the ROSE-derived balanced data (consisting of the same number of samples as the original training set, but 46.4% cases and 53.6% controls). The performance of the classifiers was assessed by the AUC value of the resulting ROC curves when the rest of the data (the other 50% of the original dataset) were used as a “testing set”. For the implementation of the methodology described above the ‘caret’ R package was used, choosing also a 4-fold cross-validation scheme repeated 1000 times. Subsequently, a recursive feature elimination algorithm, with maximum number of predictors set to 40, was used to identify an optimal subset of predictors for each of 3 best-performing classifiers chosen.

## Results

### CLL risk-related profiles

**Fig. 1:**
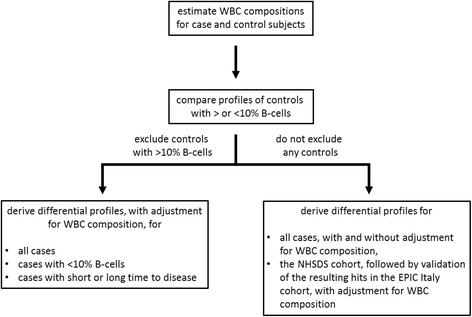
Flowchart of data analyses

First we employed the genome-wide DNA methylation profiles to estimate for each study subject the fraction of 6 major cell sub-populations (CD4-, CD8-, T- and NK cells, monocytes, granulocytes) among all WBCs. The main difference observed between case and control subjects was a large (on average 3.1fold) increase in the fraction of B-cells in cases (for details see Additional file 1: Text). As shown in Additional file 2 Table S1, a B-cell fraction >10% was a strong predictor of increased relative risks and shorter mean time to diagnosis, implying this group may have included subjects with undiagnosed MBL or CLL at recruitment. We also noted that the DNA methylation and transcriptomic profiles (adjusted for WBC composition) of control subjects with B-cell fraction >10% differed significantly from those of subjects with <10% and could not exclude the possibility that they may have been carriers, at the time of recruitment, of small clones of altered cells related to CLL (see Additional file 1: Text). Based on these observations, we opted to exclude control subjects with >10% B-cells from the derivation of all differential profiles of CLL cases discussed below (unless otherwise indicated) so as to ensure that the derived profiles reflect to the maximum degree early and mechanistically informative changes. A flowchart of the comparisons conducted using different subgroups of subjects, some of which are presented in detail in Supplementary, is shown in Fig. 1, while the numbers of differential signals obtained in the various comparisons are summarized in Additional file 2: Table S2.

#### DNA methylation profile

Comparison of the DNA methylation profiles of the CLL cases and the controls, with adjustment for WBC composition included in the statistical model, resulted in the identification of 722 differentially methylated (DM) CpG sites significant at Bonferroni-corrected *p* < 0.05 (corresponding to 494 unique genes), of which 534 showed loss of methylation in cases (mean loss 4.9%, range 0.9–30.4%), while the remaining showed methylation gain (mean gain 1.8%, range 0.1–7.9%) (Table 2 and Additional file 2: Table S3; for a discussion of the corresponding analysis without adjustment for WBC composition, as well as an assessment of the profile robustness across the two cohorts, see Additional file 1: Text). Of the 722 DM CpG sites, 530 (73.4%) overlap with 33,653 sites reported to distinguish CLL from normal B-cells [24] and show the same direction of methylation change, indicating that the majority of the DNA methylation changes which characterize our pre-diagnostic CLL risk profile are among those which accompany the transformation of normal B-cells to overt CLL clones.

**Table 2:** Top 20 CLL risk-related DM CpG sites; based on comparison of all cases vs controls with <10% B-cells, with adjustment for WBC composition

CpG site	Gene symbol	Raw *p*-value	FDR BH	LSM β, controls (%)	LSM β, cases (%)	Δβ = cases-controls (%)
cg05677184		<1E-99	1.11E-18	83.2	76.08	−7.12
cg10318725	RASA3	<1E-99	3.10E-17	87.09	79.18	−7.92
cg04308797	SEC14L1	<1E-99	2.10E-16	86.78	66.71	−20.07
cg20649847	ANKRD13B	<1E-99	4.86E-15	88.38	57.93	−30.45
cg04099036	TBCD	<1E-99	8.64E-15	87.7	74.77	−12.93
cg25212453	SLC43A2	<1E-99	8.64E-15	0.7	1.52	−10.46
cg15909319		1.06E-19	8.64E-15	87.88	77.42	0.82
cg09640070	ITPR2	<1E-99	8.33E-14	83.63	76.69	−6.94
cg19172447	EP400	<1E-99	8.33E-14	88.63	78.61	−10.01
cg06475633	P2RX1	<1E-99	3.60E-13	97.09	92.59	−4.51
cg07508446		<1E-99	4.83E-13	79.74	75.52	−4.22
cg08461425	KDM2B	<1E-99	6.63E-13	80.63	73.99	−6.64
cg05698911	DUSP22	<1E-99	1.74E-12	81.93	74.79	−7.14
cg19907483	RFX2	<1E-99	2.89E-12	90.14	83.67	−6.46
cg01595262		2.22E-16	4.10E-12	82.37	76.17	−6.2
cg21394039	ATP9B	2.22E-16	4.10E-12	85.79	79.99	−5.79
cg01438467	SLC43A2	2.22E-16	6.00E-12	89.59	83.93	−5.66
cg03777414	TVP23A	4.44E-16	9.68E-12	79.21	67.17	−12.04
cg14972228	SIPA1L3	8.88E-16	1.57E-11	82.02	77.19	−4.83
cg26363196	ST6GALNAC3	8.43E-16	1.76E-11	2.53	3.75	1.22

**Fig. 2:**
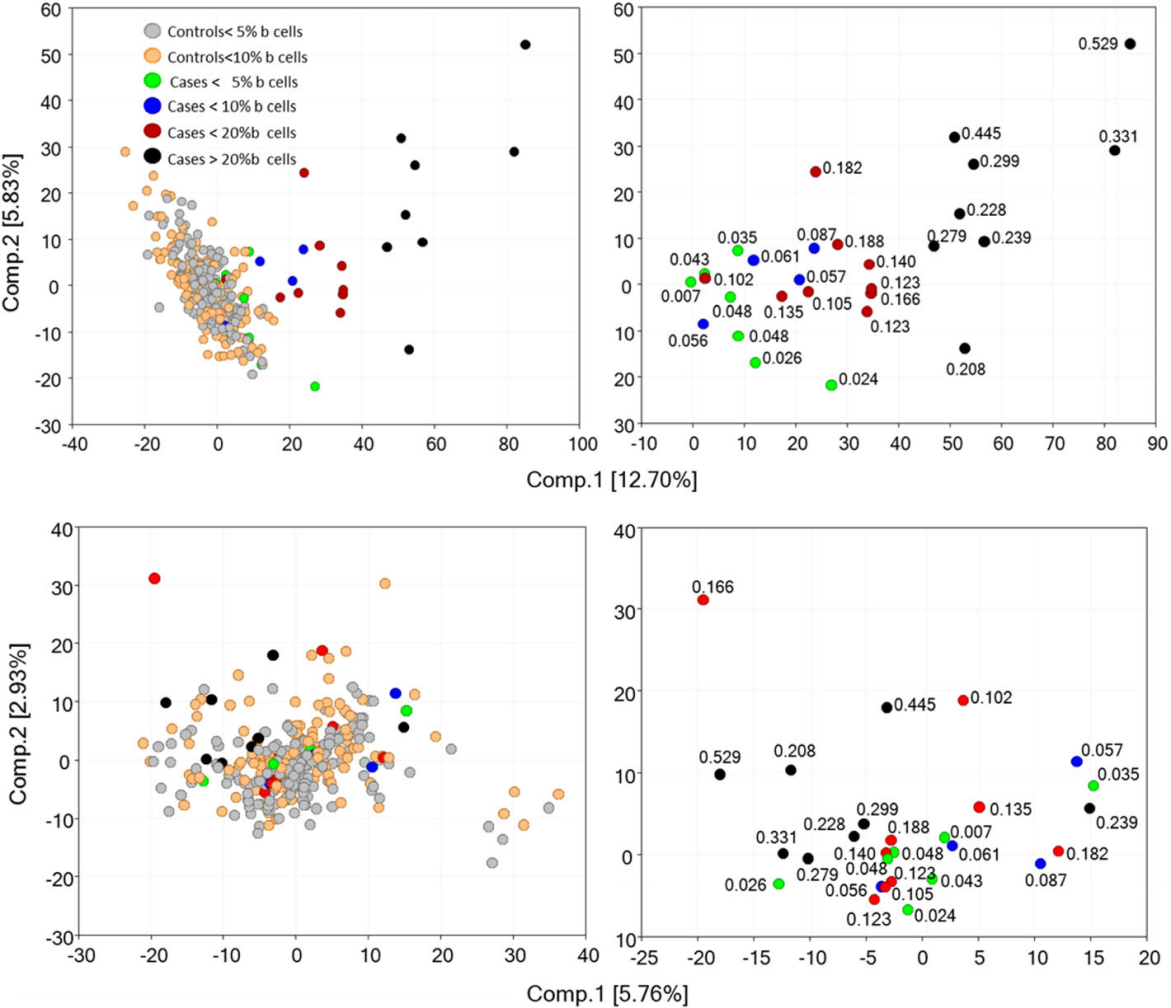
Top: PCA based on 1308 CpG sites significant (FDR < 0.05) in CLL cases and with minimal variation between WBC sub-populations. Bottom: similar analysis with 1308 CpG sites randomly selected from among those with FDR > 0.8 in CLL cases and with minimal variation between WBC sub-populations. The signal intensities employed were denoised for various parameters, including B-cell content (see Methods). The numbers in the Figures on the right indicate the fractional B-cell content of the samples. The Figures on the left show all subjects while those on the right show only the CLL case subjects

To explore the relationship of the CLL risk-related DNA methylation changes with B-cells, from the 10,785 FDR-significant DM CpGs of the CLL risk-related profile we selected those sites (1318) known to show minimal variation between different WBC subtypes [22] and performed principal component analysis (PCA) using the corresponding signal levels after denoising for batch effects, gender, cohort and smoking status (see Statistical Analysis in Additional file 1: Text). As can be seen in Fig. 2, the 28 cases are separated not only from the controls but also from each other according to their B-cell fraction. In contrast, use of an equal number of CpG sites, selected randomly from among those showing minimal variation between WBC subtypes but not included in our CLL risk-related profile, failed to yield analogous distributions. This indicates that the CLL risk-related DNA methylation signals arise in cells which carry the epigenetic hallmarks of B-cells and probably represent CLL-related B-cells.

A number of loci appear to serve as targets for extensive epigenetic modification. Thus, 176 genes and 50 intragenic CpG islands (CGIs) were represented by at least 3 (and up to 16) DM CpG sites (FDR < 0.05) each and had an enrichment (fraction of DM sites among the locus-associated sites analysed on the microarray) of at least 20% (and up to 66.7%). Furthermore, 8 of these 50 CGIs are located just upstream of the TSS1500 region of some of the same 176 genes (Additional file 2: Table S4). Gene functional classification analysis (see Bioinformatic Analysis in Additional file 1: Text) showed that the most abundant functional group among the 176 multiply DM genes consisted of 38 homeobox or homeobox-related genes, including those present in the HOX and IRX gene clusters.

#### Transcriptomic profile

Comparison of the transcriptomic profiles of 25 of our CLL cases, for which such profiles were also available, with those of control subjects (with <10% B-cells), yielded 16 differentially expressed (DE) probes, corresponding to 15 unique genes (Bonferroni-corrected *p* < 0.05; 117 probes/82 unique genes at FDR < 0.05) (Table 3 and Additional file 2: Table S5). Eleven of the Bonferroni- and 34 of the FDR-significant genes have been reported [32, 33] to be differentially expressed in B-cells isolated from CLL patients. Furthermore, 22 FDR-significant DE genes are among those found in CLL, with the same direction of change (Additional file 2: Table S5), in a meta-analysis of transcriptomic profiles of CLL patients [18]. These observations further support the suggestion that cells with changes characteristic of clinical CLL were present in our prediagnostic samples. As discussed in Supplementary, omission of adjustment for WBC composition results in a greatly increased number of significant signals, reflecting the influence of varying cell sub-populations. Nevertheless, the top 6 DE genes shown in Table 3 are the same as the top signals obtained without WBC correction (also previously reported by Chadeau-Hyam et al. [17]) and show the same direction of change. On the other hand, while the results obtained without WBC correction showed overexpression in cases of the vast majority of the significant signals, among the 117 DE signals obtained with WBC correction 51 were under-expressed in cases.

**Table 3:** CLL risk-related DE signals, Bonferroni-corrected *p* < 0.05; based on comparison of all cases vs controls with <10% B-cells, with adjustment for WBC composition

Probe ID	Gene symbol	Raw p-value	FDR BH	Fold change^a^
A_23_P500400	ABCA6	7.07E-31	4.04E-26	−5.47
A_23_P26854	ARHGAP44	1.86E-20	4.65E-16	−5.78
A_32_P53234	CEACAM21	3.48E-14	3.59E-10	−2.02
A_23_P130158	WNT3	4.34E-11	4.24E-07	−3.82
A_23_P131024	ZBTB32	3.76E-10	2.69E-06	−2.43
A_23_P27332	TCF4	8.53E-10	4.41E-06	−1.80
A_24_P691826		1.55E-09	7.79E-06	−2.33
A_23_P124335	C1orf186	6.07E-09	2.16E-05	−1.73
A_24_P29733	CDK14	6.29E-09	2.16E-05	−1.75
A_24_P306214	TLDC1	2.49E-07	6.99E-04	1.50
A_23_P85250	CD24	5.14E-07	1.41E-03	−1.60
A_23_P23639	MCOLN2	6.53E-07	1.63E-03	−1.58
A_23_P30693	PLG	9.27E-07	2.34E-03	2.39
A_32_P108156	MIR155HG	1.08E-06	2.34E-03	−1.86
A_24_P149266	PACSIN1	1.32E-06	2.34E-03	1.39
A_24_P324838	IGHD	1.43E-06	2.86E-03	1.74

^a^Positive values refer to the ratio cases/controls and negative values the ratio controls/cases

#### miRNA profile

For 11 CLL cases and 96 controls from the NHSDS cohort we were also able to examine the miRNA expression profile. We observed 2 significant signals (FDR < 0.05), miR-155-5p and miR-150-5p, both overexpressed (2.3- and 2.2fold, respectively) in cases. miR155-5p was also observed among the overexpressed genes in the transcriptomic profile, and it is notable that this gene is undermethylated in 2 out of the 6 related CpG sites in the DNA methylation profile. Both miRNAs have been reported to be overexpressed in B cells from individuals with MBL, and even more so in patients with CLL [34].

Taken together, the above results indicate that distinct epigenetic and gene expression changes, most of which are known to be associated with clinically diagnosed CLL, were present in our prediagnostic samples. Significantly, as discussed in Additional file 1: Text, we detected some of these signals also in the sub-group of cases with B-cell fraction <10% (which are less likely to have included subjects with undiagnosed CLL at recruitment) while the mean intensities of the differential signals (after adjustment for the number of B-cells) in this group were smaller than those observed in the group which includes all cases, suggesting a process of signal evolution as the size of the CLL-like cell clones increased.

### CLL risk profiles in CLL cases with <10% B-cells

In view of the evidence, discussed above, of presence in blood samples of CLL cases of clones of cells related to CLL or precursor conditions, we checked whether this was also true for cases with B-cell fraction < 10%, who are less likely to have been suffering from undiagnosed MBL or CLL at recruitment. As indicated in Additional file 2: Table S2, this subgroup (11 cases) could also be differentiated in terms of CpG methylation from the controls, although with a dramatically reduced number of signals (4 and 45 CpG sites significant at *p* < 0.05 after Bonferroni or FDR correction, respectively). All but one of these DM CpGs (Additional file 2: Table S6) are among FDR-significant CpGs observed when all cases were taken into consideration, while 25 are among those reported to distinguish CLL from normal B-cells [24]. Turning to the transcriptomic profile (Additional file 2: Table S6, lower part), 12 DE probes significant at FDR < 0.05 (4 significant at Bonferroni-corrected *p* < 0.05) were observed in low-B-cell count cases, of which 7 were also found among signals (FDR < 0.05) observed when all cases were considered, while 3 have been reported to distinguish CLL from normal B-cells [32]. No differences significant at FDR < 0.05 were observed in the miRNA profiles.

**Fig. 3:**
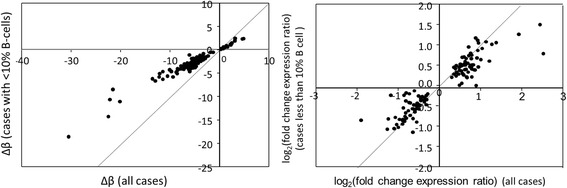
Comparison of denoised case-control methylation differences (Δβ) (left) and expression differences (foldchange ratio) (right), obtained from the comparison of all cases with controls with <10% B-cells (vertical axes) or with all controls (horizontal axes). The light lines show slope = 1

Comparison of the p-rankings of DM and DE signals observed in all cases and in those with B-cells < 10% (not shown), as well as the denoised case-control methylation differences (Δβ) or fold-change expression ratios showed strong correlations, while the mean intensities of the differences from the controls, after adjusting for B-cell content, observed in low-B-cell cases are smaller than those in all cases (Fig. 3). These observations suggest that cases with B-cell fraction <10% contained clones of cells related to CLL which evolved further towards the CLL phenotype as the CLL-like cell clones grew larger.

### Τime-To-diagnosis and CLL risk-related profiles

In view of the large variation in the time between the donation of the biological samples and clinical diagnosis of CLL (2.0–15.9 years), we derived risk-related profiles separately for subjects with time to diagnosis (TtD) longer or shorter than the median of 7.4 years. It is noted that diagnosis, as registered in the cohorts of the present study, may have corresponded to disease at different stages of progression for different case subjects, taking place in some cases accidentally during routine examination in some cases, after the appearance of clinical symptoms but not requiring treatment in others or coinciding with treatment in other subjects.

We observed 238 and 937 DM CpGs (Bonferroni-corrected *p* < 0.05) in the long and short TtD subgroups, respectively, with most (181) of the former signals being at least FDR-significant in the latter (Additional file 2: Table S7). Similarly, there were 25 and 291 DE probes (FDR *p* < 0.05) in the long and short TtD subgroups, respectively, with an overlap of 8 signals. Of the 238 DM CpGs observed in the long TtD sub-group, 168 (70.5%) have been reported to be differentially modified in clinically diagnosed CLL [24]. Furthermore, of the 21 DE genes significant in the same sub-group, 12 are among 2095 genes (hypergeometric distribution test *p* = 0.013) reported to be differentially expressed in clinical CLL [32, 33] and 9 are among those found significant in a corresponding meta-analysis included in the report by Vlaanderen et al. [18]. Finally turning to miRNA, examination of the limited number of subjects for which data were available indicates that, while no significant changes in miRNA expression could be detected in the long TtD group, 3 miRNAs (miR-155-5p, miR-150-5p, both overexpressed, and miR-4486, underexpressed) were significant (FDR < 0.05) in the short TtD group.

**Fig. 4:**
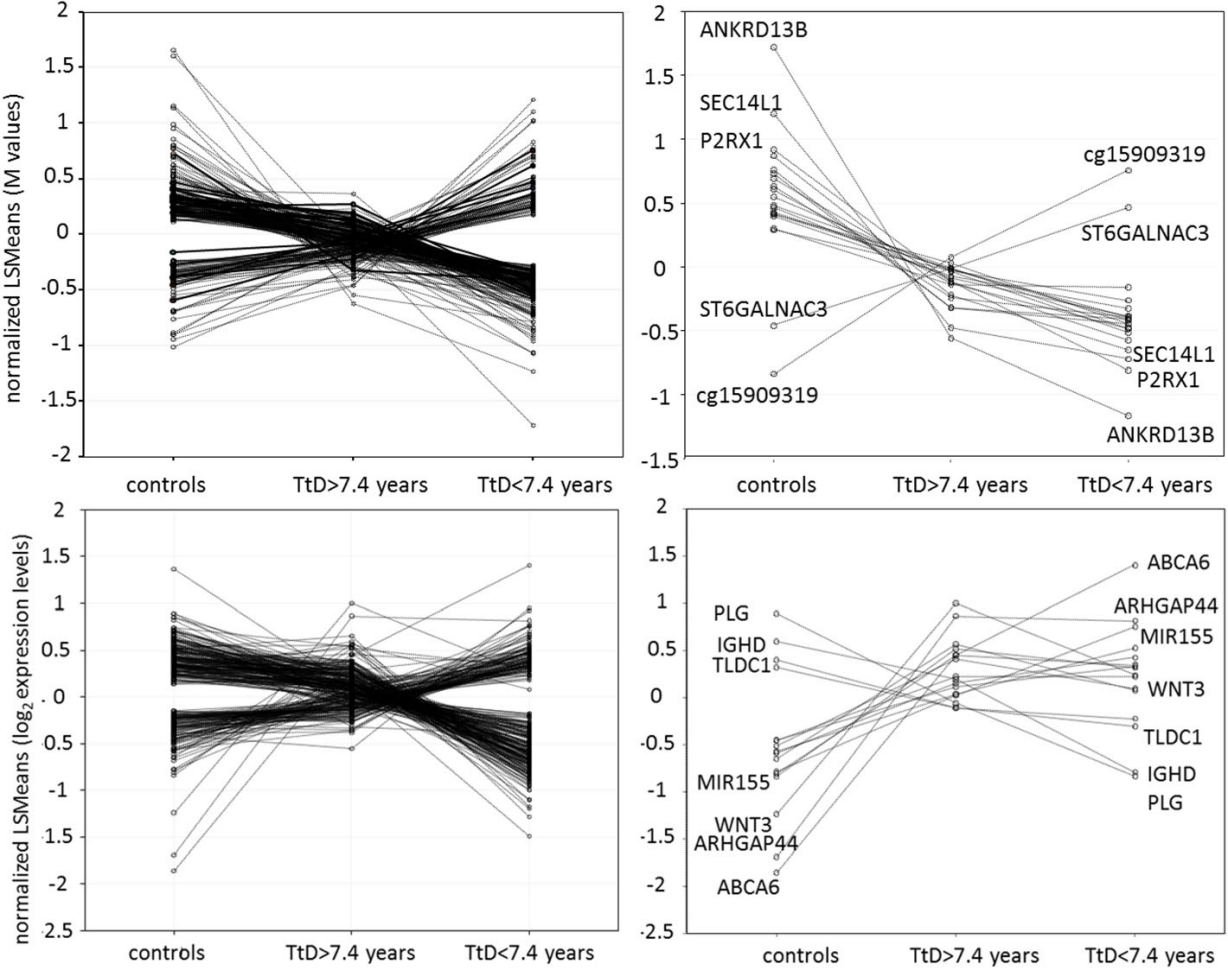
Normalized least squares means (LSM) of DM and DE signal values in the controls and the two TtD groups. Top left: 238 DM signals Bonferroni-significant in the short TtD subgroup; bottom left: 291 DE signals FDR-significant in the short TtD subgroup. Top right: top 20 DM signals observed in all cases; bottom right: Bonferroni -significant DE signals observed in all cases. In the Figures on the right are named the genes associated with the 3 DM or DE signals with the largest changes in each direction (only 2 DM genes show increased methylation with decreasing TtD)

The above observations indicate that epigenetic and gene expression changes characteristic of CLL are already present in subjects 7.4–15.7 years prior to clinical diagnosis of the disease. On the other hand, comparison of the intensities of CLL risk-related signals in control subjects and the two sub-groups with different TtD suggests the occurrence of progressive changes while approaching clinical manifestation and diagnosis of the disease. As shown in Fig. 4 (top panels), during this time the least squares means (LSM, adjusted for B-cell content – see Statistical Analysis in Supplementary) of the methylation levels of the top DM CpG sites obtained with all cases (Table 2) change in a consistent manner (mainly loss of methylation for the top 20 sites) and independently of the size of the B-cell clones. Additionally, a substantial increase in the multiplicity of gene methylation is observed in the shorter TtD group, with the average multiplicities of DM genes with at least 20% enrichment in the short TtD subgroup being 1.9 and 5.4 DM sites per gene in the long and short TtD groups, respectively (Additional file 2: Table S8). As regards the corresponding evolution of the gene expression signals (Fig. 4, bottom panels), while for most of the 16 Bonferroni-significant CLL risk-related DE signals observed in all cases expression increases in the long TtD subgroup and then remains relatively constant during the later period coming up to diagnosis, changes in both directions appear to occur, at lower statistical significance, in a large number of genes.

### Functional analysis

Gene enrichment analyses were conducted for 494 DM genes associated with DM CpGs (Bonferroni-corrected *p* < 0.05) in the CLL risk-related profile, using the recently developed BioInfoMiner web application [29] which is appropriate for the functional analysis of DNA methylation profiles (for more details see Bioinformatics analysis in Methods). For the corresponding analysis of the transcriptomic changes, in view of the small number of signals significant at Bonferoni-adjusted *p* < 0.05 (16 transcripts, 15 genes; see Table 3) which does not permit the conduct of functional analysis, we relaxed the significance criterion to FDR < 0.1. In combination with the additional statistical filters applied at the functional analysis level, such relaxation retains a strong overall statistical stringency and yields 163 DE genes. The over-represented terms thus obtained are shown in Additional file 2: Tables S9-S10. We conducted similar analyses also with the CLL risk-related gene profiles of cases with long and short TtD (Additional file 2: Tables S11-S12; no DE gene analysis was conducted for the long TtD subgroup owing to the small number of genes involved). The most notable observation is the predominance among GO terms derived from DM genes, for all sub-groups, of terms related to development and regulation of transcription as well as B-cell differentiation and physiology. Also of note is the presence of multiple DE gene-derived terms (including pathways) related to DNA damage response and *WNT* signaling.

**Fig. 5:**
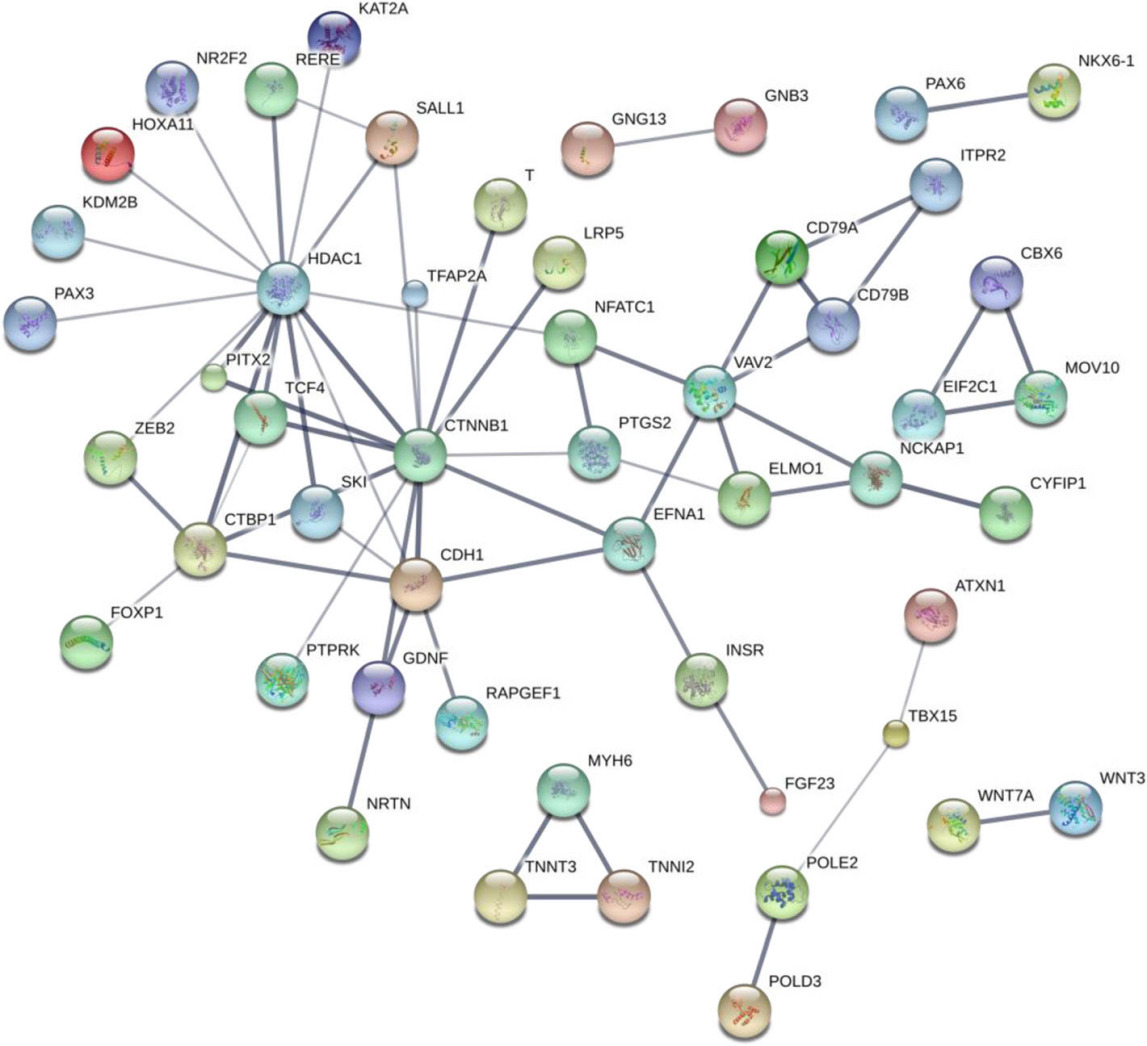
Interaction network of the DM + DE hubs (STRING)

In order to identify genes which play a central role in the biological processes leading to CLL, we used the gene prioritization module of the BioInfoMiner application to identify hub genes which are linked to multiple ontology terms, thus identifying 84 DM and 18 DE such genes (Additional file 2: Table S13). DM hubs include numerous genes that encode for transcription factors, especially members of the homeobox family (*PAX6*, multiple *HOX* genes, *FOXP1*, *EN2*, *GSC*, *EVX1*, *BARHL2*). DE hub genes include, among others, 4 genes related to WNT signaling (*WNT3*, *CTBP1*, *CTNNB1* and *TCF4*), while an additional *WNT* pathway gene, *CTBP2*, is among the DM hub genes. Examination, using the online resource Search Tool for the Retrieval of Interacting Genes (STRING) [35], of the protein-protein interaction network of the combined DM and DE hubs reveals the existence of 2 major nodes, centered on the important epigenetic modification gene *HDAC1* and the *WNT* signaling pathway gene *CTNNB1* (Fig. 5). The list of DM hub genes in the profile of long and short TtD sub-groups was also dominated by homeobox genes, while the corresponding DE genes included two genes related to *WNT* signaling, *WNT3* for the long TtD group and *CTNNB2* for the short TtD group (Additional file 2: Table S14).

### ROC analysis and development of a DNA methylation-based classification signature for predicting the development of clinical CLL

We recently reported on the advantages of using the semantics information included in the hierarchical nature of ontologies as a primary feature selection tool for the development of predictive profiles [36]. Based on this we assessed the ability of prediagnostic DM CpG sites to predict the future clinical manifestation of CLL among all subjects (i.e. without excluding case or control subjects with >10% B-cells) by focusing on 104 Bonferroni-significant DM CpG sites annotated to the hub genes. The reason for using here the profile obtained without exclusion of any of the controls is to facilitate the derivation of a predictive signature which can be of use in the general population. We employed as a training set a balanced subset corresponding to 50% of the study subjects and assessed the performance of a number of different classifiers using the remaining 50% of the population as a testing set (see ROC Analysis in Additional file 1: Text). An AUC value of 0.94 was obtained when the SVM linear model was used as a classifier. Subsequently, a recursive feature elimination algorithm, with maximum number of predictors set to 40, was used to identify an optimal subset of predictors, achieving an optimal accuracy of 95% using the Naïve Bayesian classifier and 12 predictors.

## Discussion

### B-cells with CLL-like DNA methylation and gene expression features are present in the blood more than 10 years before disease diagnosis

Using genome-wide gene expression and DNA methylation profiles observed in prediagnostic samples of whole blood leukocytes of subjects who were diagnosed with CLL 2–15.9 years later, we have derived WBC composition-corrected differential profiles which are associated with the risk of future diagnosis with CLL. Although the numbers of differential signals detected after adjustment for WBC composition are dramatically decreased relative to those found without such adjustment (Additional file 2: Table S2) [17], the majority of these signals (approx. 70% for both methylation and gene expression, both miRNAs), are known to be similarly modified in clinical CLL [18, 24, 32–34, 37]. The same holds for the signals detected in the sub-groups of cases with B-cell fraction <10% (Additional file 2: Table S6) and TtD > 7.4 years (Additional file 2: Table S7), supporting the idea that the differential risk profiles identified are unlikely to represent false findings resulting from residual confounding by variations in WBC composition but are in fact associated with the early phase of the pathogenesis of CLL. Further support for this comes from the fact that mutations in 12 of the DM genes (*FARP2, ACTA2, AcOXL, BCL2, BMF, CLPTM1L, CPEB1, CSRNP1, IPCEF1, LPP, ODF1, SERPINB6,* mostly overmethylated in cases, some at multiple CpG sites), as well as in 1 DE gene (*C11orf21*, overexpressed in cases at FDR = 0.086, have been found in GWAS studies to be associated with differential risk of CLL [38–41].

It is notable that the lists of DE and DM genes lost upon WBC adjustment overlap significantly (not shown), suggesting that their detection without WBC correction reflects variation in cell composition. Although the large impact of such correction in the present case is likely to be due to the large increase in the fraction of B-cells in CLL cases, it does underline the importance of variation in WBC composition being considered as a potential confounder not only in methylation but also in expression profiling studies of mixed cell populations [42].

The top genes in the DM and DE profiles include multiple genes related to the transport of ions and other small molecules (*SEC14L1*, *SLC43A2*, *ATP9B*, *ABC6* and *MCOLN2*), as well as genes with GTPase-related activity (*RASA3* and *TBCD*), however the role of these genes in CLL pathogenesis is not known. On the other hand, the top DE genes include 4 genes involved in *WNT* signaling - *WNT3*, *TCF4*, *CDK14*, all upregulated in cases, and *CTNNB1*, downregulated in cases (plus a fifth gene, *CTBP1*, also upregulated in cases with a statistical significance of FDR = 0.086), a key pathway in CLL (31). The DE profile also includes 2 important B-cell associated genes, *CD24*, upregulated in cases, which regulates B-cell growth, differentiation and activation and *IGHD*, downregulated in cases, the main antigen receptor on B-cells, involved in BCR signaling and the CLL-growth/survival pathway [43].

Using the significance cut-off values of Bonferroni-corrected *p* < 0.05 for DM and FDR < 0.05 for DE signals, only 1 gene (*FAM193A*, of unknown relevance to CLL) was found to be differentially both methylated and expressed in the profile obtained with WBC adjustment, supporting previous suggestions that DNA methylation is not a primary mechanism of gene regulation in CLL [24, 44].

We found two miRNAs, *miR-150-5p* and *miR-155-5p* to be consistently overexpressed in CLL cases, including the subgroups with long and short TtD. The latter gene was also differentially undermethylated at 2 CpG sites in cases. Both of these miRNAs are overexpressed in clinical CLL, the first being the most abundant miRNA in B-cells from CLL patients [34, 37]. A search on the Targetscan and miRDB databases reveals 3 of the genes which are differentially downregulated in cases to be among the targets of miR-150-5p, specifically *TLDC1* and *GLDC*, of unknown function in relation to CLL, as well as the *CTNNB1* gene which is related to *WNT* signaling, underlining the importance of this pathway in early stages of CLL pathogenesis.

To obtain a more global picture of the cellular perturbations represented by the CLL risk-related profiles, we searched for genes which are linked with multiple biological functions (hub genes) (Additional file 2: Table S13). Among the DM hub genes we identified numerous homeobox genes, including *PAX6*, multiple *HOX* genes, *FOXP1*, *EN2*, *GSC*, *EVX1* and *BARHL2*, many of which (especially *HOXA* and *HOXD*) are targets for multiple differential methylation events. *HOX* genes are involved in normal and leukemic hematopoiesis and may contribute to the mechanism of leukemic transformation [45–47], while *FOXP1* plays a particularly important role in B-cell development and enhances B-cell signaling in CLL cells [48, 49]. DE hubs include, among others, the *CBX6* gene, upregulated in cases, which is part of a polycomb complex required to maintain the transcriptionally repressed state of many genes (including *HOX*) during development [50]. Other DE hub genes include *CTBP1*, a transcriptional repressor and a key downstream component of the *WNT* signaling pathway and *NOS2*, upregulated in cases, which regulates the levels of NO which enhances the synthesis of pro-inflammatory mediators [51, 52]. The interaction network of the DM and DE hubs involves two major nodes centered upon *HDAC1* and *CTNNB1*, with a direct chain of interactions linking *HDAC1*, *CTNNB1*, *SKI*, *CTBP1* and *FOXP1* (Fig. 5). *HDAC1* is overexpressed in CLL [53] and has been shown to be instrumental in the induction of early epigenetic alterations and subsequent gene expression changes in a mouse model of CLL [54], while the SKI gene, in addition to its oncogenic and oncosuppressor functions, has been suggested to contribute to memory B cell differentiation [55].

The picture emerging from the above observations suggests the presence in our prediagnostic samples of epigenetic and expression perturbations in genes with a key role in B-cell development and differentiation. This is further supported by the results of our functional analysis, which identified GO terms which correspond mostly to cell proliferation, differentiation, developmental and regulatory processes. Of particular note is the identification of multiple ontology terms relate to B-cell physiology and morphology, as well as abnormalities of immune system physiology, such as immunodeficiency and agammaglobulinemia (Additional file 2: Table S10 and Additional file 3 Figure S1), which are characteristic clinical findings in CLL [56]. Finally, pathway analysis revealed changes in lipid and lipoprotein metabolism, Fc gamma and epsilon receptor signaling, as well as *NGF*, *VEGF*, *WNT*, *NOTCH* and B-cell signaling and DNA damage response pathways (Additional file 2: Tables S9 and S10), all of which are known to be perturbed CLL [57–62].

### CLL-related risk profiles in prediagnostic samples evolve as the B-cell clones grow and time to diagnosis decreases

The intensity of many of the early risk-related signals discussed above (especially DNA methylation-related signals) was lower in case subjects with B-cell fraction <10%, relative to that observed in all case subjects, even after adjusting for cell composition (Fig. 3). Furthermore, and while it is recognised that the use of time-to-diagnosis, rather than, e.g., time-to-first-treatment, has the limitation that diagnosis may relate to disease at varying stages of advancement, the intensity of such signals varied in a consistent manner as the time to clinical diagnosis of the disease became shorter (Fig. 4), implying that the perturbations of the cell clones in which these signals were located evolved as disease pathogenesis progressed while still remaining in a subclinical phase. Among the top risk-related epigenetic signals, DNA methylation showed mainly a pattern of decreasing levels throughout the observation period, while the top gene expression signals showed initially mainly upregulation followed by stabilization, however large numbers of signals of both types showed a consistent trend towards either over- or underexpression as the time to diagnosis became shorter (Fig. 4). Of particular interest is the evolution of *WNT3* expression, a key gene in CLL, which appeared to be upregulated in cases with long TtD and subsequently remain unchanged as TtD decreases, implying that an increase in *WNT* signaling may be an early change in CLL.

The picture of an evolving preclinical profile is also evident in the increasing multiplicity of DM CpG sites at specific loci (Additional file 2: Table S11), including 2 members of the *HOX* and 3 members of the *IRX* families which are located as clusters at different chromosomal regions. Additional file 3: Figure S2 illustrates that the tendency to accumulate increased levels of methylation at shorter TtD holds for all clusters of these genes and affects additional genes associated with these loci, such as miR196b and the *HOX* paralog genes *EVX1* and *EVX2*, as well as associated CGIs.

Focusing on DM gene hubs which are associated with long TtD (Additional file 2: Table S14) and therefore may play a role particularly in the early stages of CLL pathogenesis, the presence is noted of multiple developmentally important homeobox genes and transcription factors, implying developmental deregulation as an important part of early perturbations during CLL pathogenesis, as also indicated by the GO terms identified from the corresponding functional analysis (Additional file 2: Table S11). These genes include, among others, CSK which plays an important role in the regulation of cell growth, differentiation, migration and immune response and suppresses signaling by *BCR* [63], and *CTBP1*, involved in *WNT* and *NOTCH* signaling [64].

### A DNA methylation signature predictive of future CLL risk

We tested the ability of the set of 104 DM CpG sites associated with CLL risk-related hub genes (i.e. genes with major biological roles) to classify subjects with regard to their future risk of being diagnosed with CLL, using for this purpose a variety of classification algorithms (see ROC Analysis in Methods). An excellent predictive ability (AUC = 0.94) was obtained using all 104 of the above signals with a Naive Bayes classifier, and a similar value (AUC = 0.95) was found when just 12 predictors were selected from the above set. A high predictive ability was also observed when we used as the test set all the control samples mixed with CLL cases with <10% WBC (AUC = 0.86).

## Conclusions

We have shown that changes in CpG methylation and gene (including miRNA) expression similar to those observed in clinical CLL are present in B-cells of apparently healthy subjects 2.1–15.9 years before the diagnosis of CLL and have provided evidence supporting the idea that such changes evolve as the disease progresses from its pre-clinical stage towards clinical diagnosis. We have also shown that a CpG methylation-based signature can predict future CLL diagnosis with great accuracy. Relative to other predictive markers also reported, this predictive signature has the advantage of increased biological plausibility since it is based on hub genes many of which are known to be involved in the pathogenesis of CLL. Although patients diagnosed with cMBL or early-stage CLL do not required therapy, the great progress being made in the characterization of the genetic and epigenetic landscapes of these conditions [44, 65] raises the prospect of the development of interventions which may slow down or block progression towards full disease. In this context, early disease biomarkers that define the patients that are likely to progress to symptomatic CLL and could provide data for an individualized surveillance program, with higher intensity for some patients. Also if strong negative predictors could be defined, this could identify patients that could be reassured and not in need for follow up.

## Additional files

Additional file 1: Text. Results on WBC composition in case and control subjects, omic profiles in different subgroups of subjects and assessment of the profile robustness across the cohorts. (PDF 614 kb)

Additional file 2: Table S1. Relative risk and TtD of case sub-groups with different B-cell fraction. **Table S2.** Numbers of differential signals observed in different subject groups. **Table S3.** Top 100 DM CpGs. **Table S4.** Genes and CGI’s with at least three DM CpG sites and 20% enrichment. **Table S5.** CLL risk-related DE probes (FDR < 0.05). **Table S6.** CLL risk-associated DM and DE signals in cases with <10% B-cells compared to controls with <10% B-cells; **Table S7.** Top 100 DM and DE signals significant at FDR < 0.05 in the long and short TtD subgroups. **Table S8.** Methylation multiplicity, in the long and short TtD subgroups. **Table S9.** Ontology terms and pathways derived from the DM genes. **Table S10.** Ontology terms and pathways derived from the DE genes. **Table S11.** Ontology terms and pathways derived from the DM genes significant in different TtD subgroups. **Table S12.** Ontology terms and pathways derived from the DE genes significant in the short TtD subgroup. **Table S13.** CLL risk-related DM & DE hub genes. **Table S14.** DM and DE hub genes significant in different TtD subgroups. (XLSX 115 kb)

Additional file 3: Figure S1. The ontological tree based on DM genes. **Figure S2.** Methylation of HOX and IRX gene clusters in the short- and long-TtD subgroups. **Figure S3.** B-cell distribution in controls and CLL cases. (PDF 1281 kb)

## Abbreviations

CGI: CpG island
CLL: Chronic lymphocytic leukemia
DE: Differentially expressed
DM: Differentially methylated
LSM: Least squares means
MBL: Monoclonal B-cell lymphocytosis
NSHDS: Northern Sweden Health and Disease Study
TtD: Time-to-diagnosis
WBC: White blood cells
