# Supplementary material for: Evolving DNA methylation and gene expression markers of B-cell chronic lymphocytic leukemia are present in pre-diagnostic blood samples more than 10 years prior to diagnosis
Source: BMC Genomics. 2017 Sep 13;18:728. doi: 10.1186/s12864-017-4117-4 (PMC5598006; doi:10.1186/s12864-017-4117-4)
Supplement: Supplementary file 1 — Text. Results on WBC composition in case and control subjects, omic profiles in different subgroups of subjects and assessment of the profile robustness across the cohorts. (PDF 614 kb) [file 12864_2017_4117_MOESM1_ESM.pdf]

SUPPLEMENTARY TEXT

DNA methylation and gene expression markers of B-cell chronic lymphocytic leukemia are present in pre-diagnostic blood samples more than 10 years prior diagnosis

Panagiotis Georgiadis, Irene Liampa, Dennie G. Hebels, Julian Krauskopf, Aristotelis Chatziioannou, Ioannis Valavanis, Theo M.C.M. de Kok, Jos C.S. Kleinjans, Ingvar A. Bergdahl, Beatrice Melin, Florentin Spaeth, Domenico Palli, R. C. H. Vermeulen, J. Vlaanderen, Marc Chadeau-Hyam, Paolo Vineis and Soterios A. Kyrtopoulos, on behalf of the EnviroGenomarkers consortium

Supplementary text content

WBC composition in case and control subjects
Omic profiles in subgroups of control subjects
CLL risk-related profiles without exclusion of controls with >10% B-cells, without and with adjustment for WBC composition
Assessment of the robustness of the CLL risk-related epigenetic profile across the cohorts
References

WBC composition in case and control subjects
The mean values of WBC sub-populations, estimated as described above, are shown in Suppl. Table S15. For control subjects they are within the range reported for healthy subjects of the same age range, while the main change observed in cases is a substantial increase in the B-cell fraction ($p<0.001$; Mann-Whitney U test). B-cell fraction values showed a bell-shaped distribution in controls, in the range up to 10%, skewed to the right with 16 subjects having values in the range 10-15% (Suppl. Fig. S3). A much wider distribution was observed in cases, ranging up to 52.9% and with 17 of the 28 cases exceeding 10%. Among the other WBC subtypes, a small but statistically significant decrease was observed only in the mean fraction of granulocytes in cases, possibly reflecting the increased abundance of B-cells.

Omic profiles in subgroups of control subjects
In view of the skewed distribution of the B-cell fractions in the control subjects, and having in mind the possibility that control subjects might at enrollment already have been suffering from CLL which remained clinically undiagnosed at the time of our data collection, we compared the epigenetic profiles of control subjects with B-cell fraction >10% with those of the remaining control subjects. Because the stratification according to B-cell fraction level does not permit the inclusion of this particular variable in the statistical model employed, any DM signals thus observed may reflect the variation in B-cell fraction as well as genuine changes in methylation status. The comparison revealed 4,998 DM CpG sites (Bonferroni-adjusted $p<0.05$), 33% of which (1,666 sites; 27,792 among CpG sites significant at FDR<0.05) are known to exhibit no variation in methylation between normal WBC subtypes [1] (Suppl. Table S2) and therefore represent signals whose differential methylation cannot be attributed to variation in B-cell composition. In contrast, analogous comparison of the epigenetic profiles of control subjects with B-cell fraction <5% and 5-10% identified 1,280 DM Bonferoni-significant sites, of which only about 2% have no significant variation between normal WBC subtypes. We conclude that deviant epigenetic signals begin to emerge in large numbers in controls with B-cell fraction above approx. 10%. Comparison of the transcriptomic and the miRNA data for control subjects with B-cell fraction >10% or <10% did not reveal any differences statistically significant at FDR<0.05.

Thirtysix of the abovementioned 1,666 Bonferoni-significant DM sites are among 33,653 sites reported to distinguish CLL cells from normal B-cells [2]. Although this overlap is not statistically significant, taking into account that 1,666 is a minimum number of variant epigenetic signals potentially present in control subjects with >10% B-cells, we conclude that the possibility that these subjects may have been carriers, at the time of recruitment, of small clones of altered cells related to CLL cannot be excluded and for this reason we opted to exclude such subjects from the main analyses of CLL-related profiles presented in the main text. However, for reasons of completeness, and to enable comparison with previously published data, we also derived, and discuss below, the corresponding profiles using data from all control subjects.

CLL risk-related profiles without exclusion of controls with >10% B-cells, without and with adjustment for WBC composition
<u>Epigenetic profile:</u> Comparison of the epigenomic profiles of the 28 CLL cases with those of the 319 controls, initially without adjustment for WBC composition, showed significant (Bonferoni-corrected $p<0.05$) differences in 12,065 CpG sites. Inclusion in the statistical model of adjustment for WBC composition led to the loss of more than 90% of these signals, implying that the signals lost were associated with variation in WBC composition, and the vast majority of the signals (94.2%, see Suppl. Table S2) thus obtained overlapped with those obtained when using all control subjects for the comparison. Suppl. Table S16 shows the top 100 epigenetic signals obtained using all controls and WBC adjustment, all of which are also significant (Bonferoni-corrected $p<0.05$) in the profile obtained with exclusion of controls with >10% B-cells.

<u>Transcriptomic profile:</u> For 25 of the 28 CLL cases and 282 of the controls of the present study, genome-wide gene expression profiles were also available. We therefore went on to compare these profiles with those of control subjects, without and with adjustment for WBC composition. The profile obtained without adjustment for WBC composition consists of 595 differentially expressed (DE) probes significant at

Bonferoni-corrected p<0.05 (5,286 at FDR<0.05; Suppl. Table S2) and extensively overlaps with the transcriptomic profile we reported previously based on a larger number of cases (N=39) from the same study [3]. Adjusting for WBC composition led to loss of the vast majority of these signals, yielding 34 DE probes (Bonferoni-corrected p<0.05; 305 at FDR <0.05) (Suppl. Table S2). A large fraction of the DE genes lost upon adjustment for WBC composition were also among the DM genes lost after the corresponding adjustment of the epigenetic profiles (data not shown), supporting the idea that their variation between cases and controls reflects differences in WBC composition rather than genuine changes in their expression or epigenetic status. Suppl. Table S17 shows the 117 transcriptomic signals with FDR<0.05 obtained using all controls and WBC adjustment, 114 of which are also significant (FDR<0.05) in the profile obtained with exclusion of controls with >10% B-cells.

miRNA profile

For 11 CLL cases and 100 controls from the Swedish cohort we were also able to examine the miRNA expression profile. Both with and without adjustment for WBC composition we observed the same 2 significant signals (FDR<0.05) as observed when excluding controls with >10% B-cells, namely miR-155-5p and miR-150-5p, over-expressed in cases.

Assessment of the robustness of the CLL risk-related epigenetic profile across the cohorts

In view of the relatively limited numbers of subjects available, to evaluate the robustness of the observed profiles between the two population cohorts employed in our study, we restricted the intial (discovery) analysis to the NSHS cohort and used the EPIC Italy cohort as a validation cohort. Comparison of the 19 CLL cases versus the 184 controls of the NSHDS cohort (including all controls regardless of B-cell content), with adjustment for WBC composition, resulted in 557 CpG sites significant at Bonferroni-corrected p<0.05, of which 415 (75%) are among the corresponding 722 signals observed in the mixed population (Suppl. Table S2). Subsequent examination, in a case vs control comparison, of these 557 CpG sites in the EPIC Italy cohort resulted in 264 (47%) DM CpGs significant at the FDR<0.05 level. Based on this overlap between the two cohorts, and having in mind a) the differences in the mean B-cell fractions between the CLL cases of the two cohorts (EPIC Italy: 11%, SD 0.11 ; NSHS: 18%, SD 0.14 ; Mann-Whitney U test p=0.1) and b) the particularly small number of cases in the EPIC-ITALY cohort, we conclude that the two cohorts give similar results and for this reason the main data analysis was conducted only with the pooled cohort.

References:

1. Houseman EA, Accomando WP, Koestler DC, Christensen BC, Marsit CJ, Nelson HH, et al. DNA methylation arrays as surrogate measures of cell mixture distribution. BMC Bioinformatics. 2012;13:86.

2. Kulis M, Heath S, Bibikova M, Queirós AC, Navarro A, Clot G, et al. Epigenomic analysis detects widespread gene-body DNA hypomethylation in chronic lymphocytic leukemia. Nat. Genet. 2012;44:1236–42.

3. Chadeau-Hyam M, Vermeulen RCH, Hebels DG a. J, Castagné R, Campanella G, Portengen L, et al. Prediagnostic transcriptomic markers of Chronic lymphocytic leukemia reveal perturbations 10 years before diagnosis. Ann. Oncol. Off. J. Eur. Soc. Med. Oncol. 2014;25:1065–72.
