# Supplementary material for: Evolving DNA methylation and gene expression markers of B-cell chronic lymphocytic leukemia are present in pre-diagnostic blood samples more than 10 years prior to diagnosis
Source: BMC Genomics. 2017 Sep 13;18:728. doi: 10.1186/s12864-017-4117-4 (PMC5598006; doi:10.1186/s12864-017-4117-4)
Supplement: Supplementary file 3 — The ontological tree based on DM genes. Figure S2. Methylation of HOX and IRX gene clusters in the short- and long-TtD subgroups. Figure S3. B-cell distribution in controls and CLL cases. (PDF 1281 kb) [file 12864_2017_4117_MOESM3_ESM.pdf]

SUPPLEMENTARY FIGURES

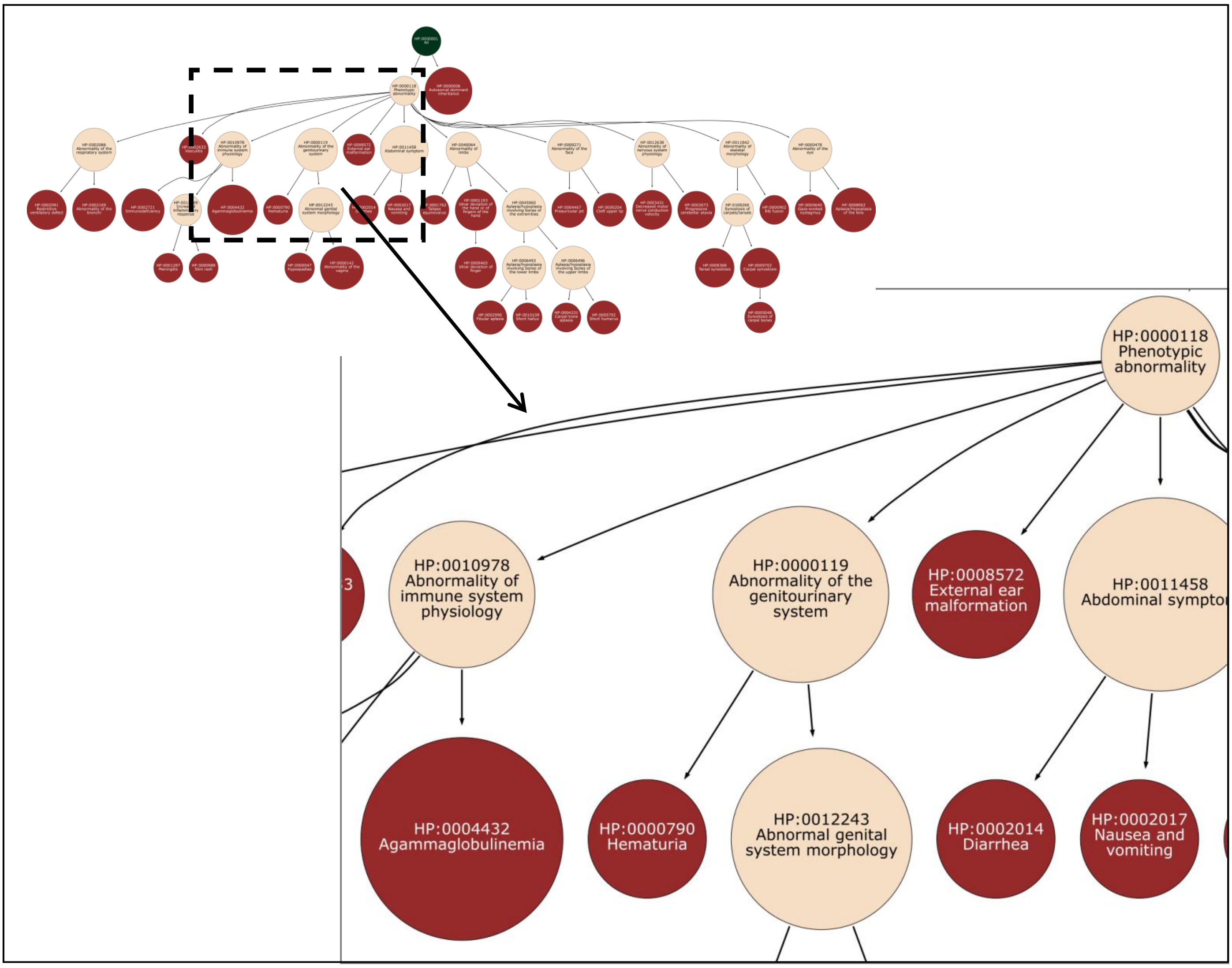

Suppl. Fig. S1: The ontological tree, based on HPO, of significant terms (red) derived from the DM genes. The dashed line outlines terms related to immune system physiology. Linkage depicts the hierarchical clustering of terms, based on the Resnik semantic similarity metric.

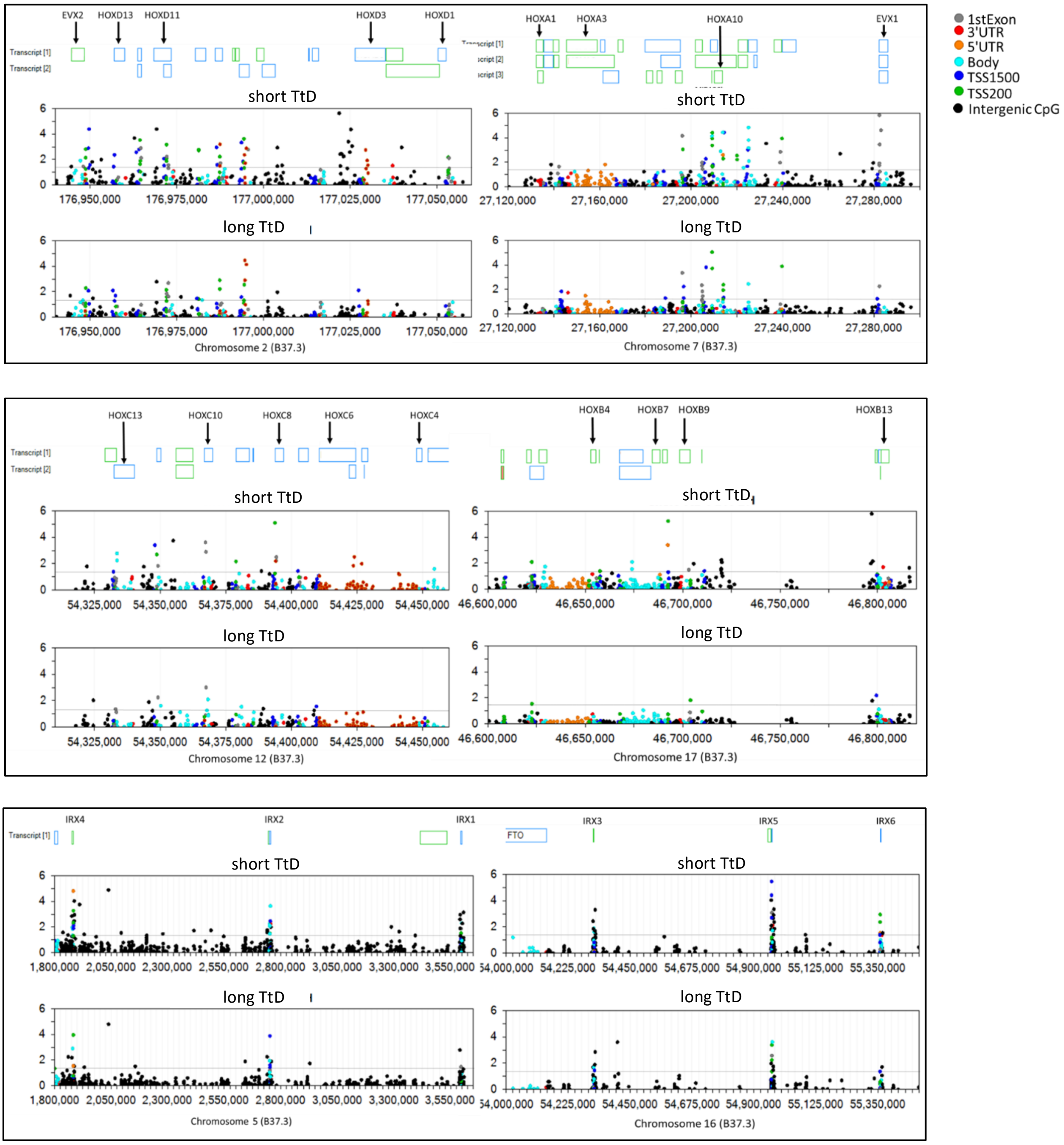

Suppl. Fig. S2: Methylation maps of HOX and IRX gene clusters in different TtD subgroups. The horizontal axes denote the chromosomal locations of αλλ CpG sites and the vertical axes the -log(FDR) values for the corresponding case-control differences. Top left: HOXD cluster; top right: HOXA cluster; middle left: HOXC cluster; middle right: HOXB cluster; bottom left and right: IRX clusters. The thin horizontal lines indicate the limits of FDR-significant signals

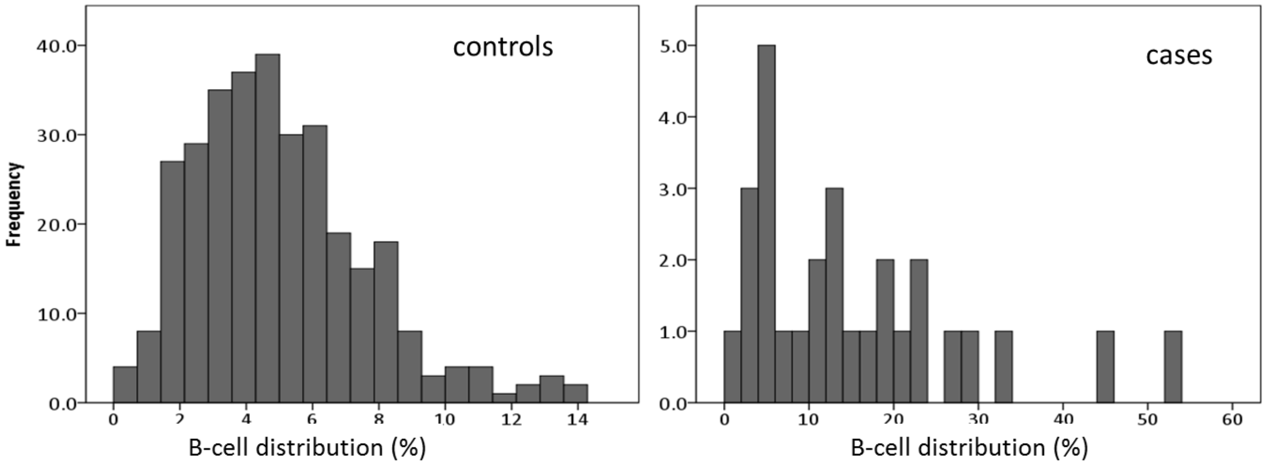

Suppl. Fig. S3: B-cell distribution in controls (left) and CLL cases (right)
